# Effects of Cooling Temperatures via Thermal K2P Channels on Regeneration of High-Frequency Action Potentials at Nodes of Ranvier of Rat Aβ-Afferent Nerves

**DOI:** 10.1523/ENEURO.0308-21.2021

**Published:** 2021-09-15

**Authors:** Hirosato Kanda, Sotatsu Tonomura, Jianguo G. Gu

**Affiliations:** 1 Department of Anesthesiology and Perioperative Medicine, School of Medicine, University of Alabama at Birmingham, Birmingham, AL 35294; 2 Department of Pharmacology, Hyogo University of Health Sciences, Kobe, Hyogo 650-8530, Japan

**Keywords:** Aβ-afferent nerves, action potential, cooling temperature, node of Ranvier, saltatory conduction, two-pore domain K^+^ channels

## Abstract

Temperature-sensitive two-pore domain potassium channels (thermal K2P) are recently shown to cluster at nodes of Ranvier (NRs) and play a key role in action potential (AP) regeneration and conduction on Aβ-afferent nerves. Cooling temperatures affect AP regeneration and conduction on Aβ-afferent nerves but the underlying mechanisms are not completely understood. Here, we have performed patch-clamp recordings directly at the NR in an *ex vivo* trigeminal nerve preparation. We have characterized the effects of cooling temperatures on intrinsic electrophysiological properties and AP regeneration at the NR on rat Aβ-afferent nerves, and determined whether and how thermal K2P channels may be involved in the effects of cooling temperatures. We show that cooling temperatures from 35°C to 15°C decrease outward leak currents, increase input resistance, depolarize resting membrane potential (RMP), broaden AP width and increase latency of AP threshold at the NR. We further demonstrate that cooling temperatures impair regeneration of high-frequency AP trains at the NR. The effects of cooling temperatures on the intrinsic electrophysiological properties and regeneration of high-frequency AP trains at the NR can be partially reversed by BL-1249 (BL), arachidonic acid (AA), and intra-axonal protons, three thermal K2P activators, indicating the involvement of thermal K2P channels. Moreover, we show that at cooling temperatures there are interplays among thermal K2P channels, RMPs, and voltage-gated Na^+^ channels, which together limit regeneration of high-frequency AP trains at the NR. Our findings demonstrate a new role of thermal K2P channels in temperature-dependent conduction of high-frequency sensory signals.

## Significance Statement

Cooling temperatures impair high-frequency saltatory conduction along Aβ-afferent nerves to compromise tactile acuity. Here, we have performed patch-clamp recordings directly at the node of Ranvier (NR) of rat trigeminal Aβ-afferent nerves to determine how cooling temperatures affect high-frequency saltatory conduction. We show that cooling temperatures via thermal K2P channels alter intrinsic electrophysiological properties and impair regeneration of high-frequency AP trains at the NR. Moreover, we demonstrate that there are interplays among thermal K2P channels, resting membrane potentials (RMPs) and voltage-gated Na^+^ channels at cooling temperatures to limit regeneration of high-frequency action potential (AP) trains at the NR. Our findings establish a new role of thermal K2P channels in temperature-dependent conduction of somatosensory signals, which may have implications in cooling-induced sensory dysfunctions and analgesic effects of cooling.

## Introduction

Action potential (AP) propagation along myelinated nerves, known as saltatory (hop or leap in Latin) conduction, is achieved by APs leaping along myelinated nerves via the nodes of Ranvier (NRs; Ranvier, 1871, 1872; Lillie, 1925; Tasaki, 1939; Huxley and Stampfli, 1949; Boullerne, 2016). Saltatory conduction can reach speeds over 100 m/s and frequencies over hundreds of hertz. In somatosensory nerves, saltatory conduction along Aβ-afferent nerves conveys sensory information essential for tactile tasks such as social interaction, environmental exploration, and tactile discrimination. High-speed conduction of APs along Aβ-afferent nerves ensures rapid sensory responses. On the other hand, high-frequency AP trains conducted along Aβ-afferent nerves are essential for sensory acuity (Phillips and Matthews, 1993). Saltatory conduction can be profoundly affected by cooling temperatures. Most previous studies have focused on effects of cooling temperatures on AP conduction velocities (Franz and Iggo, 1968; Cabanes et al., 2003; Stecker and Baylor, 2009). Cooling temperatures are also known to affect regeneration and conduction of high-frequency AP trains (Paintal, 1965; Franz and Iggo, 1968), but this effect has received less attention. Interestingly, it has been shown in humans that cooling temperatures can impair regeneration of high-frequency AP trains on afferent nerves leading to poor tactile discrimination (Phillips and Matthews, 1993).

Cooling temperatures can directly slow kinetics and reduce amplitudes of voltage-activated Na^+^ currents (Matteson and Armstrong, 1982; Sarria et al., 2012). This is thought to be a major contributing factor by which cooling temperatures can impair regeneration of high-frequency AP trains on nerves including Aβ-afferent fibers. In addition to directly affecting voltage-gated Na^+^ channels, cooling temperatures may suppress the activity of other ion channels to alter intrinsic electrophysiological properties of membranes at the NR. This may also be an important contributing factor causing impairment of regeneration of high-frequency AP trains on Aβ-afferent fibers. However, this hypothesis has never been directly tested because of technical challenges in the application of patch-clamp recording technique aimed to study ion channels and their functions at the NR.

Immunohistochemical studies have shown that voltage-gated Na^+^ channels Nav1.6 and Nav1.2 are expressed at mammalian NRs (Caldwell et al., 2000; Waxman, 2006). Immunohistochemical studies also have shown that voltage-gated K^+^ channels Kv7.2 (Devaux et al., 2004) and Kv3.1b (Devaux et al., 2003) are present at mammalian NRs, and Kv1.1 as well as Kv1.2 are present in juxtaparanodal regions of myelinated axons (Rhodes et al., 1997). Mechanisms underlying assembly of these ion channels and other proteins at the NR have been gradually understood (Rasband and Peles, 2021). Previous studies have failed to detect significant voltage-activated K^+^ currents at intact NRs in myelinated nerves of mammals (Chiu and Ritchie, 1980), suggesting that voltage-gated K^+^ channels located at and near the NR are functionally silent. This raises the question of how APs can be repolarized to allow regeneration of APs at the NR. Only recently have we found that TREK-1 and TRAAK channels are clustered at the NR of rat myelinated nerves, functioning as principal K^+^ channels for AP repolarization (Kanda et al., 2019). Another recent study also reports wide expression of TRAAK channels at the NR of myelinated nerves of mice (Brohawn et al., 2019).

TREK-1 and TRAAK belong to the two-pore domain K^+^ (K2P) channel family which mediate “leak” or “background” K^+^ currents across cell membranes. K2P channels, distinct from voltage-gated K^+^ channels (Goldstein et al., 2005; Lotshaw, 2007; Enyedi and Czirják, 2010; Viatchenko-Karpinski et al., 2018), are involved in setting resting membrane potentials (RMPs) and membrane input conductance (Enyedi and Czirják, 2010). K2P channels play roles in regulation of neuronal excitability, sensory transduction, and nociception (Lotshaw, 2007). Mutations of these channels result in abnormal functions of central and peripheral nervous systems in humans (Bauer et al., 2018; Royal et al., 2019). Interestingly, TREK-1 and TRAAK are highly temperature sensitive and can be substantially inhibited by cooling temperatures, and thus are termed thermal K2P channels (Maingret et al., 1999, 2000; Kim et al., 2001; Kang et al., 2005; Enyedi and Czirják, 2010; Viatchenko-Karpinski et al., 2018). TREK-1 and TRAAK clustered at NRs of Aβ-afferents play a key role in AP repolarization, and high-speed and high-frequency saltatory conduction (Kanda et al., 2019). Furthermore, TREK-1 and TRAAK channels are involved in cooling temperature-induced reduction of AP conduction velocities on Aβ-afferent nerves (Kanda et al., 2019). Since cooling temperatures also affect regeneration and conduction of high-frequency AP trains (Paintal, 1965; Franz and Iggo, 1968), the aforementioned findings (Kanda et al., 2019) raise the question as whether and how thermal K2P channels may be involved in impairment of high-frequency AP trains on Aβ-afferent nerves at cooling temperatures. In the present study, we set out to address this question by performing patch-clamp recordings directly at the NR of Aβ-afferent fibers in *ex vivo* preparations of rat trigeminal nerves.

## Materials and Methods

### *Ex vivo* trigeminal nerve preparation

Adult Sprague Dawley rats (Envigo) of both sexes were used in this study. Unless otherwise indicated, animals included in the present study were at the ages of five to seven weeks. The rats were housed in a temperature-controlled animal facility (23°C) and maintained on a 12/12 h light/dark cycle. Animal care and use conformed to NIH guidelines, and experimental protocols for animal use were approved (IACUC-21 486) by the Institutional Animal Care and Use Committee at the University of Alabama at Birmingham.

Rats were euthanized by overdose of isoflurane followed by decapitation, and trigeminal nerve bundles (∼15 mm) with their ganglions were dissected out and placed in a Petri dish filled with ice cold Leibovitz’s L-15 medium (Corning Cellgro). Under a dissection microscope, connective tissues on the surface of the nerve bundles were carefully removed with a pair of fine forceps. Trigeminal nerve bundles with their ganglions were then affixed in a recording chamber by a tissue anchor and submerged in a Krebs’ solution that contained the following: 117 mm NaCl, 3.5 mm KCl, 2.5 mm CaCl_2_, 1.2 mm MgCl_2_, 1.2 mm NaH_2_PO_4_, 25 mm NaHCO_3_, and 11 mm glucose. The Krebs’ solution was saturated with 95% O_2_ and 5% CO_2_, had pH adjusted to 7.35 with NaOH and osmolarity adjusted to 324 mOsm with sucrose, and maintained at room temperature of 24°C. The recording chamber was mounted on the stage of an Olympus BX51 microscope equipped with IR-DIC and fluorescent imaging systems. To facilitate the penetration of patch-clamp electrodes through perineural tissues, the nerve bundles were briefly exposed to a mixture of 0.07% Dispase II (Roche) and 0.07% collagenase (MilliporeSigma) in Krebs’ solution for 5 min at the room temperature, and the enzymes were then washed off with Krebs’ solution. Unless otherwise indicated, the *ex vivo* trigeminal nerve preparation was continuously perfused with Krebs’ bath solution at 24°C.

### Pressure-clamped patch-clamp recordings at the NR

NRs in myelinated nerves of *ex vivo* trigeminal nerve preparations were visualized under a 40× water immersion objective (NA 0.80) and with an infrared CCD camera (IR-1000, DAGE-MTI). Myelinated nerves chosen for all experiments had diameters (including myelin thickness) of 8–10 μm. These myelinated nerves were Aβ-afferent nerve fibers based on conduction velocity (Kanda et al., 2019). Patch-clamp recordings were applied to the NR of trigeminal Aβ-afferent nerve fibers. In brief, recording electrodes were fabricated using a Flaming/Brown Micropipette Puller (P-97, Shutter Instruments). The electrode resistance after filling recording electrode internal solutions were 8–10 MΩ. For all experiments, the recording electrode was filled with an internal solution containing the following: 105 mm K-gluconate, 30 mm KCl, 0.5 mm CaCl_2_, 2.4 mm MgCl_2_, 5 mm EGTA, 10 mm HEPES, 5 mm Na_2_ATP, and 0.33 mm GTP-TRIS salt; the pH of the solution was adjusted to 7.35 with KOH. The recording electrode was connected with a high-speed pressure-clamp device (HSPC-1; ALA Scientific Instruments) to control electrode internal pressure. A high positive pressure of 200 mmHg was first applied via the recording electrode to pressure-clean the surface areas around the NR. Intraelectrode positive pressures were then reduced to 90 ± 10 mmHg, while the recording electrode penetrated the perineurium wrapped on nodal axons. Once the recording electrode penetrated through the perineurium, intraelectrode pressure was reduced to 5 mmHg to approach nodal axons. Optimally accessing nodal axon membranes was judged by the reduction of seal-test currents and the appearance of a small current oscillation. Once the electrode tip optimally assessed nodal membranes, intraelectrode positive pressure was gradually reduced and a pressure of −6 ± 2 mmHg was applied into recording electrode until forming gigaohm seals (usually >5 GΩ) between the recording electrode and nodal axon membranes. To achieve whole-cell configuration, nodal membranes were ruptured by a train of short electrical pulses (±200 mV, 20 ms each pulse) delivered through the patch-clamp recording electrode while intraelectrode pressure was held at a constant negative pressure of −30 mmHg. After establishing whole-cell mode, negative pressure was reduced to −5 mmHg and maintained during recordings. Signals of voltage-clamp experiments were recorded and amplified using an Axopach 200B amplifier or a multiclamp 700B amplifier, filtered at 2 kHz and sampled at 10 kHz using the pCLAMP 10 software (Molecular Devices). Signals of current-clamp recordings for APs at the NR were low-pass filtered at 2 kHz and sampled at 50 kHz.

To determine intrinsic electrophysiological properties including both passive and active properties of nodal membranes, patch-clamp recordings were performed under whole-cell current-clamp configuration. Step current pulses were injected into the NR through the recording electrode. Step currents ranged from −100 to 1800 pA with increments of 50 pA per step, and the duration of each step was 1 s. Unless otherwise indicated, membrane potentials mentioned in texts have been corrected for the calculated junction potential of 12 mV.

To determine AP conduction at the NR of myelinated afferent nerves, APs were evoked at a peripheral end of the infraorbital nerve branch using a suction stimulation electrode. The suction stimulation electrode’s tip size was ∼1 mm in diameter and was fire-polished. The peripheral end of trigeminal afferent nerve (infraorbital branch) was aspirated into the suction stimulation electrode with a tight fitting by negative pressure. The negative pressure was continuously applied into the suction stimulation electrode to maintain the tight fitting during experiments. To initiate a train of APs at the peripheral end of the nerve, monophasic square wave pulses were generated by an electronic stimulator (Master-8, A.M.P.I.) and delivered via a stimulation isolator (ISO-Flex, A.M.P.I.) to the suction stimulation electrode. The duration of each stimulation pulse was 50 μs. Minimum stimulation intensity for evoking APs, i.e., stimulation threshold at the peripheral end of the nerves was first determined and then train stimulation was applied at the intensity of 2-fold threshold (3.52 ± 0.34 mA, *n* = 12) throughout the experiments. To determine success rates of APs at the NR following different frequencies of train, stimulation pulses were applied to the trigeminal nerve bundles at frequencies of 1, 10, 50, 100, 200, 500, and 1000 Hz. Stimulation train duration was 20 s, and intervals between stimulation trains were 30 s. Success rates of APs regenerated at the NR were the percentage of successfully regenerated APs at the nodal recording sites during 20-s train stimulation.

### AP-clamp recordings at the NR

Pressure-clamped patch-clamp recordings were applied to the NR of Aβ-afferent nerves in *ex vivo* trigeminal nerves obtained from animals at the ages of 9–12 weeks. APs at the NR were first recorded under the current clamp configuration following electrical stimulation at peripheral ends of trigeminal nerve bundles. For AP-clamp experiments (Llinás et al., 1982; Liu et al., 2017), recordings were performed under the voltage-clamp configuration and each NR’s own AP was used as the voltage command waveform. AP waveforms (AP train stimulations) were applied at frequencies of 10, 50, and 100 Hz for 20 s. AP-clamp experiments were performed with nodal membranes held at −75 and −84 mV, and experiments were performed at temperatures of 15°C and 35°C. AP-evoked Na^+^ currents were quantified by integrating net inward current during the rising phase of AP, starting at the baseline and continuing to the peak of AP wave forms.

### Effects of temperatures and K2P channel activators

Effects of temperatures on intrinsic electrophysiological properties of nodal membranes as well as AP regeneration at the NR of trigeminal Aβ-afferent nerves were determined by the aforementioned electrophysiological experiments at Krebs’ bath solution temperatures of 35°C, 24°C, and 15°C. The temperatures of bath solutions were controlled by a Peltier temperature control system (CL-200A, Warner Instrument), and were continuously monitored with a thermal probe placed in the recording chamber (TA-29, Warner Instrument). The bath solution was applied at 2 ml/min from a short tube (500 μm in internal diameter) whose outlet was positioned 1 cm away from the recording site. The time was <1 min for warming from 24°C to 35°C and <2 min for cooling from 24°C to 15°C at the recording site.

Intrinsic electrophysiological properties of the NR were examined in the absence and presence of BL-1249 (BL; 10 μm, Tocris Bioscience), arachidonic acid (AA; 20 μm, Tocris Bioscience), or protons (intracellular low pH 5). These reagents were shown to potentiate leak K^+^ channel activity at NRs (Kanda et al., 2019). These reagents were either applied through bath solution to the recording chambers (BL) or applied intracellularly through recording electrode internal solutions (AA, protons). For pharmacology tests with bath application of BL, it was perfused to *ex vivo* nerve preparations for 10 min through a pinch valve drug delivery system (VC-6, Warner Instrument). For experiments with intracellular applications of testing compounds, each compound was dissolved in recording electrode internal solutions and recordings were performed 20 min after establishing whole-cell configuration.

### Data analysis

Electrophysiological data were measured using Clampfit 10 (Molecular Devices). Data collected from trigeminal nerves of 16 male and 11 female animals were aggregated for data analysis since no significant differences in electrophysiological results were found between male and female animals. Outward Leak currents were measured at the membrane holding voltage of −72 mV. Input resistance was determined with a 10-mV voltage step from the membrane holding voltage of −72 mV. RMPs were measured under current-clamp configuration at the zero holding current. AP rheobase was the threshold step current that evoked AP firing. AP threshold was the threshold potential at which AP upstroke started. Latency to AP was the time from the beginning of the step current to AP threshold. AP amplitude or upstroke was measured from AP threshold to AP peak. AP width was measured as the duration from 50% AP upstroke to 50% AP repolarization. All the above AP parameters were determined with the AP evoked by the rheobase step current at the NR. AP success rate at the NR was calculated as the number of APs recorded at the NR divided by the number of electrical stimuli, where the electrical stimuli were applied to the peripheral ends of trigeminal nerves at different frequencies for 20 s. Curve-fitting was performed using a nonlinear regression fit with the following equation: Y = 100/(1 + 10^((LogIC50-X)*HillSlope))), where Y is normalized response (AP success rate), X is variable (time or stimulation frequency), IC50 is time to 50% of success rate (T_50_) or stimulation frequency at which success rate is 50% (FS_50_). All data analyses were performed using GraphPad Prism (version 7). Unless otherwise indicated, all data are reported as mean ± SEM of *n* independent observations. Statistical significance was evaluated using one-way ANOVA with Tukey’s *post hoc* tests or Student’s *t* tests. Differences were considered to be significant with *p <* *0.05, **p <* *0.01, ***p <* *0.001, and not significant (ns) with *p *≥* *0.05.

## Results

### Cooling temperatures alter intrinsic electrophysiological properties of the NR on Aβ-afferent nerves

**Figure 1.**
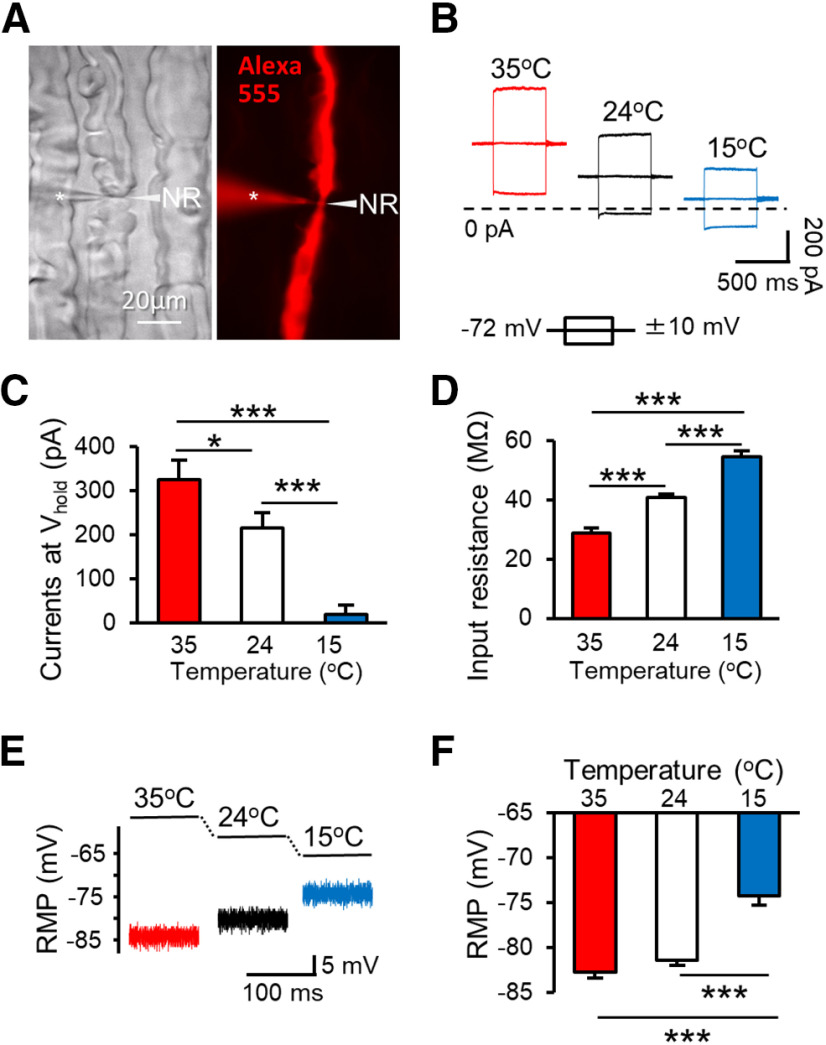
Cooling temperatures alter passive membrane properties of the NR on Aβ-afferent nerves. ***A***, left panel, bright field image shows Aβ-afferent nerves in an e*x-vivo* trigeminal afferent nerve preparation. Right panel, fluorescent image shows patch-clamp recordings at a NR of a trigeminal Aβ-afferent nerve. The electrode (asterisk indicated) contained Alexa Fluor 555 to trace the NR (arrow indicated) and other axonal regions. ***B***, Three sets of sample traces recorded at 35°C (left), 24°C (middle), and 15°C (right) show outward leak currents at the holding potential of −72 mV and membrane responses to ±10-mV voltage steps. Dashed line indicates the level of 0-pA current. ***C***, Summary data of the outward leak currents recorded at the NR at 35°C (*n* = 8), 24°C (*n* = 8), and 15°C (*n* = 8). The outward leak currents were determined at the holding potential (V_hold_) of −72 mV. ***D***, Summary data of the input resistance of nodal membranes determined at 35°C (*n* = 11), 24°C (*n* = 17), and 15°C (*n* = 14). The input resistance was calculated based on the current following a 10-mV voltage step. ***E***, Sample traces show nodal RMPs at 35°C, 24°C, and 15°C. ***F***, Summary data of the nodal RMP at 35°C (*n* = 11), 24°C (*n* = 17), and 15°C (*n* = 14). Data represent mean ± SEM, *p <* *0.05, **p <* *0.01, ***p <* *0.001, one-way ANOVA with the Tukey’s *post hoc* test.

We have hypothesized that cooling temperatures via thermal K2P channels alter intrinsic electrophysiological properties of the NR to affect regeneration of high-frequency AP trains on Aβ-afferent fibers. To test this idea, we first determined effects of cooling temperatures on both passive and active membrane properties at the NR of Aβ-afferent nerves in our *ex vivo* trigeminal nerve preparations by using the pressure-clamped patch-clamp recordings at the NR (Kanda et al., 2019, 2021). Figure 1*A* illustrates a pressure-clamped patch-clamp recording experiment performed at an NR of a large diameter trigeminal afferent nerve. In this example, the axon was labeled intracellularly by the fluorescent dye Alexa Fluor 555 included in recording electrode internal solution (Fig. 1*A*). We performed recordings at NRs of myelinated trigeminal afferent nerves with outer diameters including myelin sheath ∼10 μm. We recently reported trigeminal afferent nerve fibers with diameters ∼10 μm were Aβ-afferent nerves based on conduction velocity (Kanda et al., 2019). In the present study, we found at NRs of Aβ-afferent nerves, membrane leak currents at the holding potential of −72 mV were outward, and progressively reduced with decreasing temperature from 35°C to 24°C and 15°C (Fig. 1*B*). This is consistent with leak K^+^ currents mediated by the thermal K2P channels TREK-1 and TRAAK that are expressed at NRs of these afferent nerves as shown in our recent study (Kanda et al., 2019). The leak K^+^ currents at holding potential of −72 mV were 324.9 ± 44.1 pA (*n* = 8) at 35°C, decreased to 216.1 ± 34.7 pA (*n* = 8, *p* < 0.05) at 24°C, and further decreased to 18.9 ± 20.1 pA (*n* = 8, *p* < 0.001) at the cooling temperature of 15°C (Fig. 1*C*). Reduction of leak K^+^ currents was accompanied by increases of nodal membrane input resistance with cooling temperatures (Fig. 1*D*). Nodal membrane input resistances were obtained at the holding potential of −72 mV based on the membrane responses to ±10 mV membrane testing (Fig. 1*B*). The input resistance was 28.8 ± 1.7 MΩ (*n* = 11) when measured at 35°C (Fig. 1*B*,*D*). As temperature was reduced, progressive increase in nodal membrane input resistance was observed. For example, nodal membrane input resistance was significantly increased to 40.8 ± 1.1 MΩ (*n* = 17, *p* < 0.01) at 24°C, and further increased to 54.5 ± 2.1 MΩ (*n* = 14, *p* < 0.01) at 15°C (Fig. 1*D*). Importantly, accompanying reduced leak K^+^ currents and increased nodal membrane input resistance, RMPs measured at NRs were progressively depolarized at cooling temperatures. At 35°C, the RMP at NRs was at a hyperpolarized level of −82.7 ± 0.6 mV (*n* = 11, Fig. 1*E*,*F*), close to the reversal potentials of K^+^ channels under our experimental conditions. The RMPs were significantly depolarized to −74.2 ± 1 mV (*n* = 14, *p* < 0.001) at 15°C (Fig. 1*E*,*F*). These results may suggest that cooling temperatures via inhibition of thermal K2P channels significantly shifted RMPs to depolarized levels at the NR.

**Figure 2.**
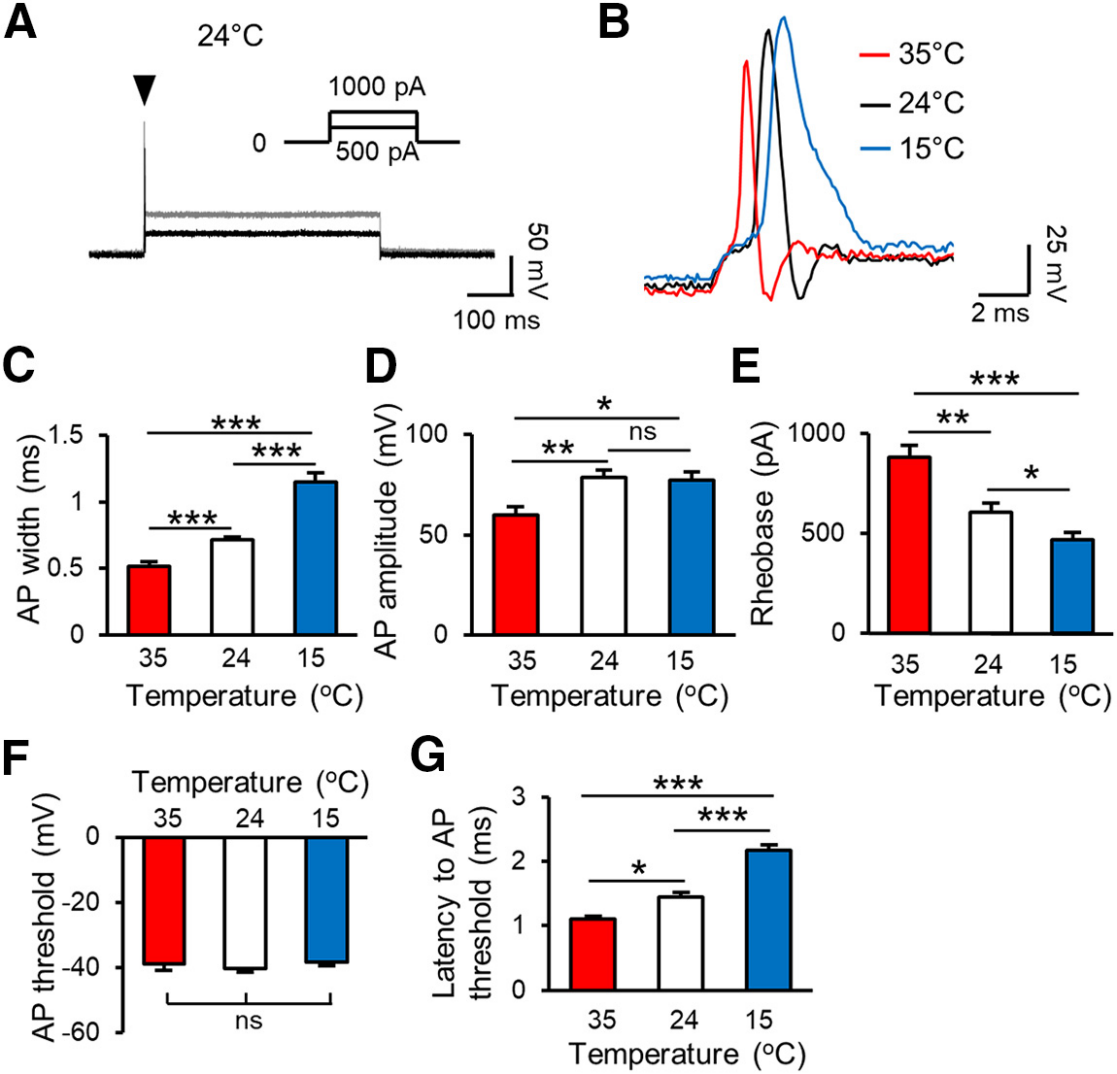
Cooling temperatures alter active membrane properties at the NR. ***A***, Sample traces show APs (arrowhead indicated) recorded at a NR in response to step currents (inset) injected via recording electrode into the NR. The recording was performed under whole-cell current-clamp configuration at 24°C. ***B***, Three overlay sample traces at expanded scale show APs recorded at an NR at 35°C (red), 24°C (black), and 15°C (blue). APs were evoked by rheobase step currents injected through recording electrode into the NR. ***C–G***, Summary data at 35°C (*n* = 11), 24°C (*n* = 17), and 15°C (*n* = 14) of AP width (***C***), amplitude or upstroke (***D***), rheobase (***E***), threshold (***F***), and latency to AP threshold (***G***). Data represent mean ± SEM, *p <* *0.05, **p <* *0.01, ***p <* *0.001, one-way ANOVA with the Tukey’s *post hoc* test. ns, not significant.

We next determined effects of cooling temperatures on properties of APs at the NR. In this set of experiments, recordings were performed at the NR under whole-cell current-clamp configuration and APs were elicited by injecting step currents through the recording electrode (Fig. 2*A*). APs evoked by step currents at 35°C, 24°C, and 15°C showed different kinetics as assessed by AP width (Fig. 2*B*,*C*). AP width was 0.52 ± 0.03 ms (*n* = 11) at 35°C, and prolonged to 0.72 ± 0.02 ms (*n* = 17, *p* < 0.001) at 24°C, and further prolonged to 1.15 ± 0.07 ms (*n* = 14, *p* < 0.001) at 15°C (Fig. 2*C*). Other significant changes in AP properties include amplitude (Fig. 2*D*), rheobase (Fig. 2*E*), and latency to AP threshold (Fig. 2*G*). AP amplitude or AP upstroke was 59.8 ± 4.2 mV (*n* = 11) at 35°C, and increased to 78.9 ± 3.6 mV (*n* = 17, *p* < 0.05) at 24°C and 77.3 ± 4.3 mV (*n* = 14, *p* < 0.001) at 15°C (Fig. 2*D*). AP rheobase was 881.8 ± 60 pA (*n* = 11) at 35°C, and decreased to 605.9 ± 48.7 pA (*n* = 17, *p* < 0.01) at 24°C and 471.4 ± 32.6 pA (*n* = 11, *p* < 0.001) at 15°C (Fig. 2*E*). AP threshold was −38.9 ± 1.9 mV (*n* = 11) at 35°C, and was not significantly altered at 24°C and 15°C (Fig. 2*F*). At AP rheobase level, latency to AP threshold was 1.1 ± 0.05 ms (*n* = 7) at 35°C, and significantly prolonged to 1.4 ± 0.07 ms (*n* = 7, *p* < 0.05) at 24°C and 2.2 ± 0.09 ms (*n* = 7, *p* < 0.001) at 15°C (Fig. 2*G*).

### Thermal K2P activators partially counteract cooling temperature-induced changes of intrinsic electrophysiological properties of the NR

**Figure 3.**
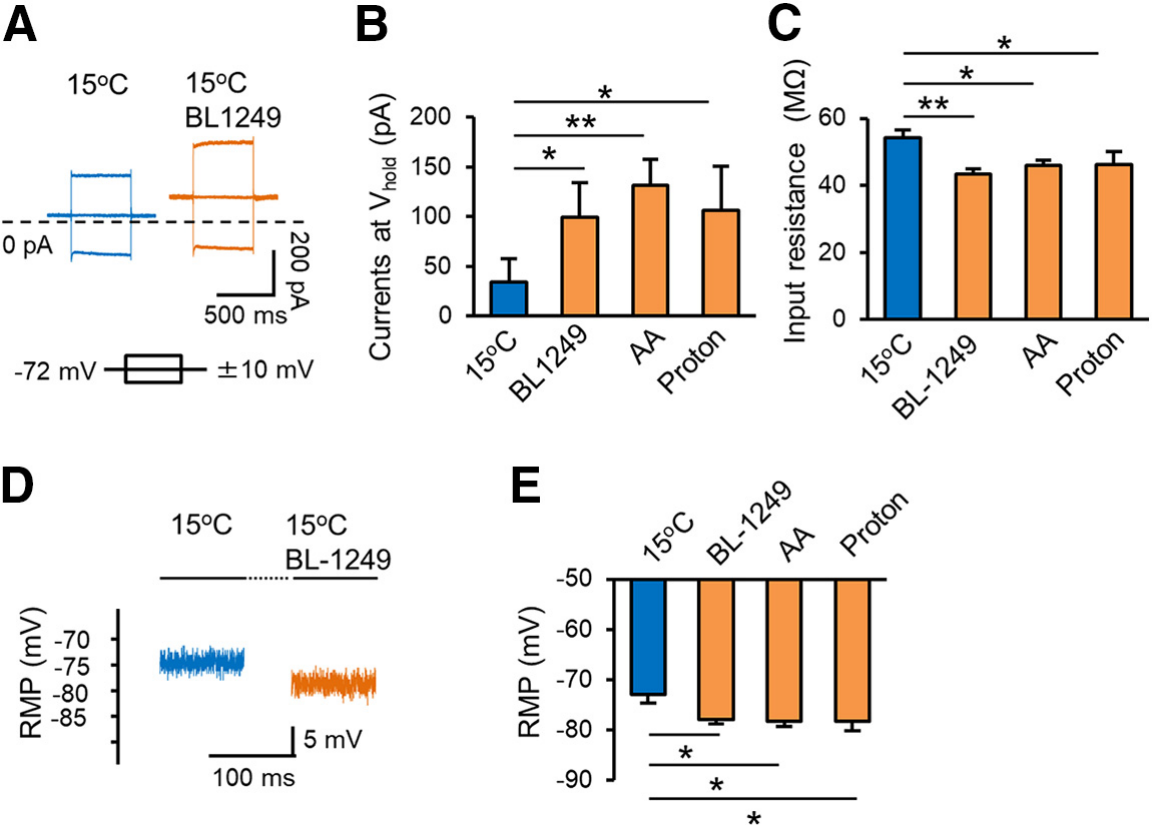
Thermal K2P activators partially counteract cooling temperature-induced changes of passive membrane properties of the NR. ***A***, Two sets of sample traces show outward leak currents and membrane responses to testing pulses of ± 10-mV voltage steps at cooling temperature of 15°C in the absence (left) and presence the thermal K2P activator BL (right). Nodal membranes were held at −72 mV. Dashed line indicates the level of 0-pA current. ***B***, Summary data of outward leak currents determined at the NR at 15°C in the absence (*n* = 6), presence of thermal K2P activators BL (10 μm, *n* = 9), AA (20 μm, *n* = 8), and protons ([pH]_i_ = 5, *n* = 8). Nodal membranes were held at −72 mV. ***C***, Summary date of nodal membrane input resistance at 15°C in the absence (*n* = 6), presence of 10 μm BL (*n* = 9), 20 μm AA (*n* = 8), and protons ([pH]_i_ = 5, *n* = 8). ***D***, Sample traces show RMPs at the NR at 15°C in the absence (Blue) and presence of 10 μm BL (orange). ***E***, Summary data of RMP at 15°C in the absence (*n* = 6), presence of 10 μm BL (*n* = 7), 20 μm AA (*n* = 9), and protons ([pH]_i_ = 5, *n* = 8). In all experiments, BL was bath applied, AA and protons ([pH]_i_ = 5) were applied intracellularly through recording electrode internal solution. Data represent mean ± SEM, *p <* *0.05, **p <* *0.01, one-way ANOVA with the Tukey’s *post hoc* test.

To support the idea that cooling temperature-induced changes of passive membrane properties at NRs were via suppressing thermal K2P channel activity, we investigated whether the effects of cooling temperatures could be reversed by thermal K2P activators. We have recently shown that the activity of thermal K2P channels at NRs can be potentiated by BL, AA, and intra-axon protons, three activators of thermal K2P channels (Enyedi and Czirják, 2010; Kanda et al., 2019). Therefore, we examined passive membrane properties at 15°C in the absence and presence of these activators. While cooling temperatures from 35°C to 15°C decreased leak K^+^ current, increased input resistance and depolarized RMP at the NR (Fig. 1*D–F*), the effects of cooling temperatures were partially reversed by BL, AA, and protons (Fig. 3*A–D*). For example, the leak K^+^ current at holding potential of −72 mV was 34.3 ± 23 pA (*n* = 7) at 15°C in the absence of thermal K2P activators, and increased to 99.3 ± 34.6 pA (*n* = 7, *p* < 0.05) in the presence of 10 μm BL, 131.1 ± 26.4 pA (*n* = 9, *p* < 0.01) in the presence of 20 μm AA, and 106.3 ± 43.9 pA (*n* = 8, *p* < 0.05) in the presence of protons ([pH]_i_ = 5; Fig. 3*B*). The input resistance was 54.2 ± 2.5 MΩ (*n* = 6) at 15°C in the absence of thermal K2P activators, and decreased to 43.3 ± 1.7 MΩ (*n* = 9, *p* < 0.01) in the presence of 10 μm BL, 45.9 ± 1.7 MΩ (*n* = 8, *p* < 0.05) in the presence of 20 μm AA, and 46.3 ± 3.8 MΩ (*n* = 8, *p* < 0.05) in the presence of protons ([pH]_i_ = 5, Fig. 3*C*). The RMP was −73.0 ± 1.6 mV (*n* = 6) at 15°C in the absence of thermal K2P activators, and became more negative at −77.9 ± 0.9 mV (*n* = 7, *p* < 0.05) in the presence of 10 μm BL, −78.3 ± 0.9 mV (*n* = 9, *p* < 0.05) in the presence of 20 μm AA, and −78.3 ± 1.9 mV (*n* = 8, *p* < 0.05) in the presence of protons ([pH]_i_ = 5, Fig. 3*D*,*E*). These results suggest that thermal K2P channels are involved in cooling temperature-induced changes of the passive membrane properties at the NR.

**Figure 4.**
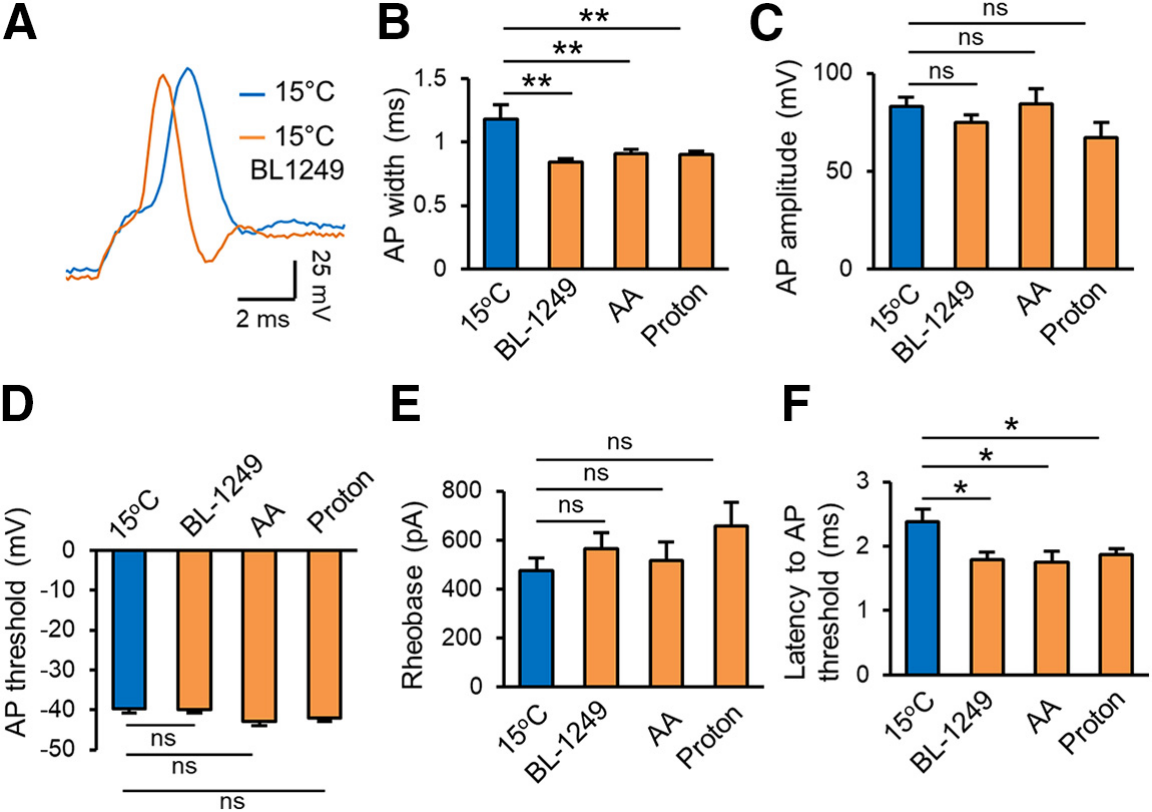
Effects of thermal K2P activators on active membrane properties at the NR at cooling temperatures. ***A***, Two overlay sample traces of APs recorded at a NR at cooling temperature of 15°C in the absence (blue) and presence of 10 μm BL (orange). APs were evoked by rheobase step currents injected through recording electrode into the NR. ***B–F***, Summary data of active membrane properties at 15°C in the absence (*n* = 6), present of 10 μm BL (*n* = 8), 20 μm AA (*n* = 7), and protons ([pH]_i_ = 5, *n* = 8). Active membrane properties measured at the NR include AP width (***B***), amplitude (***C***), threshold (***D***), rheobase (***E***), and latency to AP threshold (***F***). Data represent mean ± SEM; ns, not significantly different, *p <* *0.05, **p <* *0.01, one-way ANOVA with the Tukey’s *post hoc* test.

To determine whether effects of cooling temperatures on active membrane properties at NRs were also mediated by thermal K2P channels, we tested effects of thermal K2P activators on AP properties at NRs at cooling temperatures. While cooling temperature of 15°C significantly prolonged AP width (Fig. 2*C*), AP width at 15°C was shortened significantly in the presence of BL, AA, or protons (Fig. 4*B*). For example, AP width was 1.18 ± 0.02 ms (*n* = 6) at 15°C in the absence of thermal K2P activators, and shortened to 0.84 ± 0.02 ms (*n* = 8, *p* < 0.01) in the presence of 10 μm BL, 0.91 ± 0.03 ms (*n* = 7, *p* < 0.01) in the presence of 20 μm AA, and 0.9 ± 0.03 ms (*n* = 8, *p* < 0.01) in the presence of protons ([pH]_i_ = 5; Fig. 4*B*). AP amplitude, threshold, and rheobase were not significantly affected by these K2P activators (Fig. 4*C–E*). At the rheobase level, latency to AP threshold was significantly shortened by the K2P activators (Fig. 4*F*). The latency was 2.4 ± 0.2 ms (*n* = 6) at 15°C in the absence of thermal K2P activators, and shortened to 1.78 ± 0.12 ms (*n* = 8, *p* < 0.05) in the presence of 10 μm BL, 1.75 ± 0.17 ms (*n* = 6, *p* < 0.05) in the presence of 20 μm AA, and 1.87 ± 0.1 ms (*n* = 6, *p* < 0.05) in the presence of protons ([pH]_i_ = 5; Fig. 4*F*). These results suggest that thermal K2P channels are involved in cooling temperature-induced changes of some active membrane properties at the NR.

### Cooling temperatures suppress and thermal K2P activators partially rescue the regeneration of high-frequency AP trains at the NR

**Figure 5.**
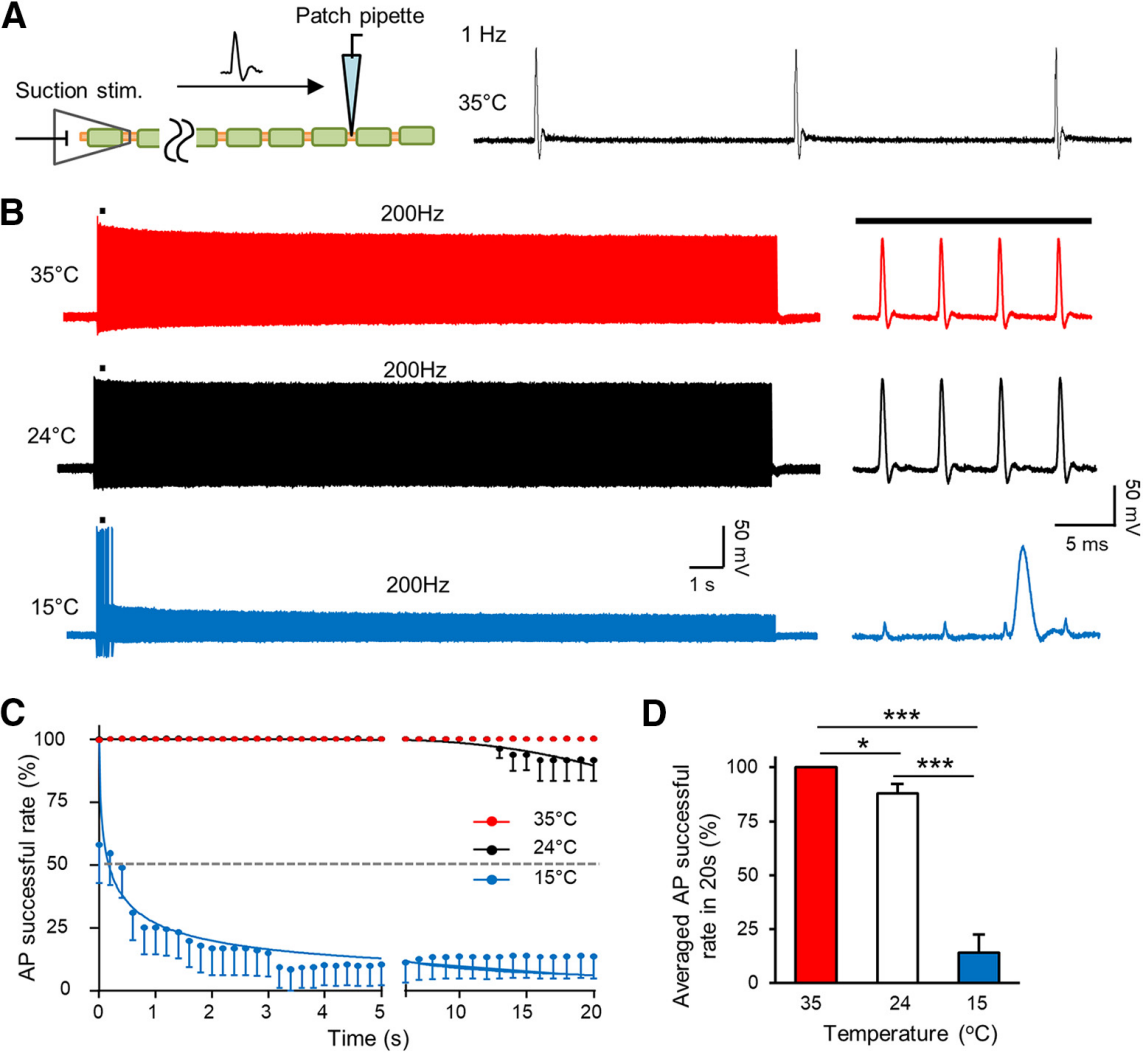
Cooling temperatures reduce AP success rate at the NR in response to 200-Hz train stimulation. ***A***, Schematic diagram illustrates experimental setting (left) and sample traces of APs evoked by electrical stimulation and recorded at a NR at 35°C (right). Electrical stimulation was applied at 1 Hz at a distal site of the nerve bundle. ***B***, Three sets of sample traces show APs recorded at 35°C (top), 24°C (middle), and 15°C (bottom) in response to 200-Hz train stimulation for 20 s. Traces on right are at expanded scale. ***C***, Time course of AP success rates in response to 200-Hz train stimulation for 20 s with experiments conducted at 35°C (red circles, *n* = 6), 24°C (black circles, *n* = 6), and 15°C (blue circles, *n* = 6). Time bin: 200 ms. ***D***, Bar graph shows averaged AP success rate at 35°C (*n* = 6), 24°C (*n* = 6), and 15°C (*n* = 6) in response to the 200-Hz train stimulation for 20 s. Data represent mean ± SEM, *p <* *0.05, ***p <* *0.001, one-way ANOVA with the Tukey’s *post hoc* test.

We have previously shown that APs can be regenerated at the NR with high success rates at 24°C in response to train stimulation at frequencies up to 200 Hz. (Kanda et al., 2019). In the present study, we investigated effects of cooling temperatures on success rates of AP regeneration at the NRs, and the involvement of thermal K2P channels in changes of AP success rate. In this set of experiments, APs were elicited by train stimulation applied to a distal end of the nerve bundle, and APs were recorded at a NR that was ∼15 mm away from the stimulation site (Fig. 5*A*). As shown in Figure 5*B–D*, at 35°C, Aβ-afferent fibers followed 200-Hz train stimulation with AP success rate near 100% during the course of 20-s train stimulation. At 24°C, AP success rate remained nearly 100% for up to 12 s and slightly decreased in the remaining 8 s of 200-Hz train stimulation (Fig. 5*B*,*C*). In contrast, at the cooling temperature of 15°C and with 200-Hz train stimulation, AP success rate rapidly dropped to <25% in <2 s (Fig. 5*B*,*C*). The APs mostly failed to regenerate in the remaining time of the 20-s train stimulation at 15°C (Fig. 5*C*). On average for the train stimulation at 200 Hz for 20 s, AP success rate was 100 ± 0% (*n* = 6) at 35°C, slightly reduced to 88 ± 4.3% (*n* = 6, *p* < 0.05) at 24°C, and largely reduced to 14 ± 8.6% (*n* = 6, *p* < 0.001) at the cooling temperature of 15°C (Fig. 5*D*).

**Figure 6.**
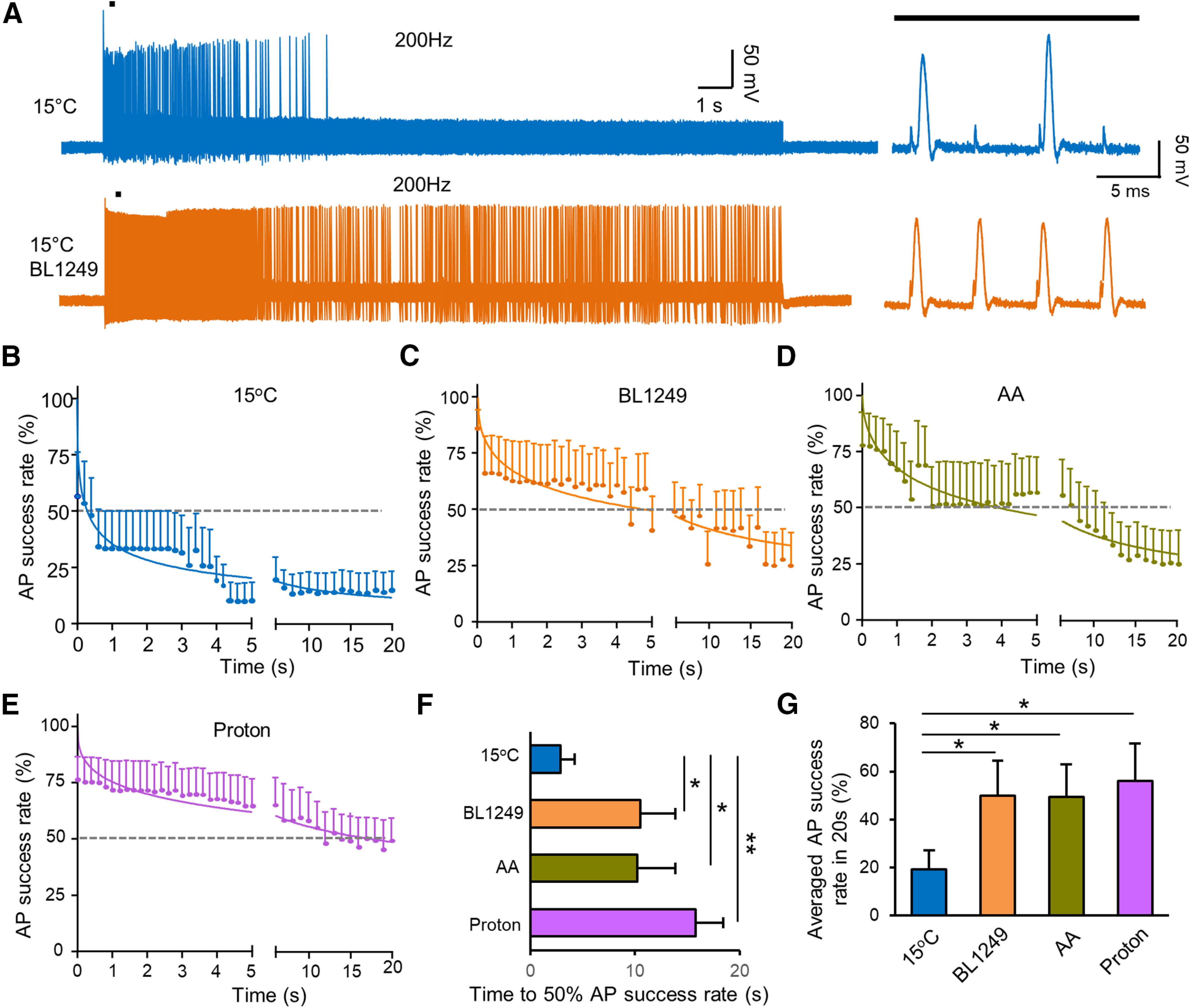
Thermal K2P activators partially counteract cooling temperature-induced reduction of AP success rate in response to 200-Hz train stimulation. ***A***, Two sets of sample traces show nodal APs evoked by 200-Hz train stimulation for 20 s in the absence (top), and presence (bottom) of 10 μm BL. Experiments were performed at 15°C. Traces on the right are at an expanded scale. ***B–E***, Time course of success rate of nodal APs over 20 s in the absence (***B***, *n* = 6), presence of 10 μm BL (***C***, *n* = 6), 20 μm AA (***D***, *n* = 6), or protons (***E***, [pH]_i_ = 5, *n* = 6). Time bin: 200 ms. Dashed line in each panel indicates 50% AP success rate. ***F***, Summary data of the time at which nodal AP success rate at 15°C reduced to 50% (T_50_) in the absence (control, *n* = 6), present of 10 μm BL (*n* = 6), 20 μm AA (*n* = 6), and protons ([pH]_i_ = 5, *n* = 6). ***G***, Summary data of averaged success rate of nodal APs in 20 s at 15°C in the absence (control, *n* = 6), presence of 10 μm BL (*n* = 6), 20 μm AA (*n* = 6), and protons ([pH]_i_ = 5, *n* = 6). Data represent mean ± SEM, *p <* *0.05, **p <* *0.01, ***p <* *0.001, one-way ANOVA with the Tukey’s *post hoc* test.

To test the idea that thermal Kv2P channels at NRs are involved in the reduction of AP success rates at cooling temperatures, we investigated whether thermal K2P activators could reverse the effects of cooling temperatures on AP success rate. In this set of experiments, APs elicited with 200-Hz train stimulation for 20 s were recorded at NRs at 15°C in the absence, or presence of BL, AA or protons. As shown in Figure 6*A*,*B*, AP success rate in response to 200-Hz train stimulation rapidly decreased over time at 15°C in the absence of thermal K2P activators (Fig. 6*A*,*B*). However, AP success rate was less reduced at 15°C in the presence of 10 μm BL (Fig. 6*A*,*C*), 20 μm AA (Fig. 6*D*), or protons ([pH]_i_ = 5; Fig. 6*E*). We used two parameters to quantitatively describe the effects of the thermal K2P activators on AP success rate following 200-Hz train stimulation, the time at which AP success rate reduces to 50% (T_50_; Fig. 6*F*) and average AP success rate during 20-s train stimulation (Fig. 6*G*). At 15°C, T_50_ was 2.87 ± 1.38 s (*n* = 6) in the absence of thermal K2P activators, and significantly prolonged to 10.5 ± 3.33 s (*n* = 6, *p* < 0.05) in the presence of 10 μm BL, 10.2 ± 3.6 s (*n* = 6, *p* < 0.05) in the presence of 20 μm AA, and 15.7 ± 2.68 s (*n* = 6, *p* < 0.01) in the presence of protons ([pH]_i_ = 5; Fig. 6*F*). On average, AP success rate was 19.2 ± 7.8% (*n* = 6) in the absence of thermal K2P activators, and significantly increased to 49.9 ± 14.5% (*n* = 6, *p* < 0.05) in the presence of 10 μm BL, 49.4 ± 13.7% (*n* = 6, *p* < 0.05) in the presence of 20 μm AA, and 56 ± 15.6% (*n* = 5, *p* < 0.05) in the presence of protons ([pH]_i_ = 5; Fig. 6*G*).

**Figure 7.**
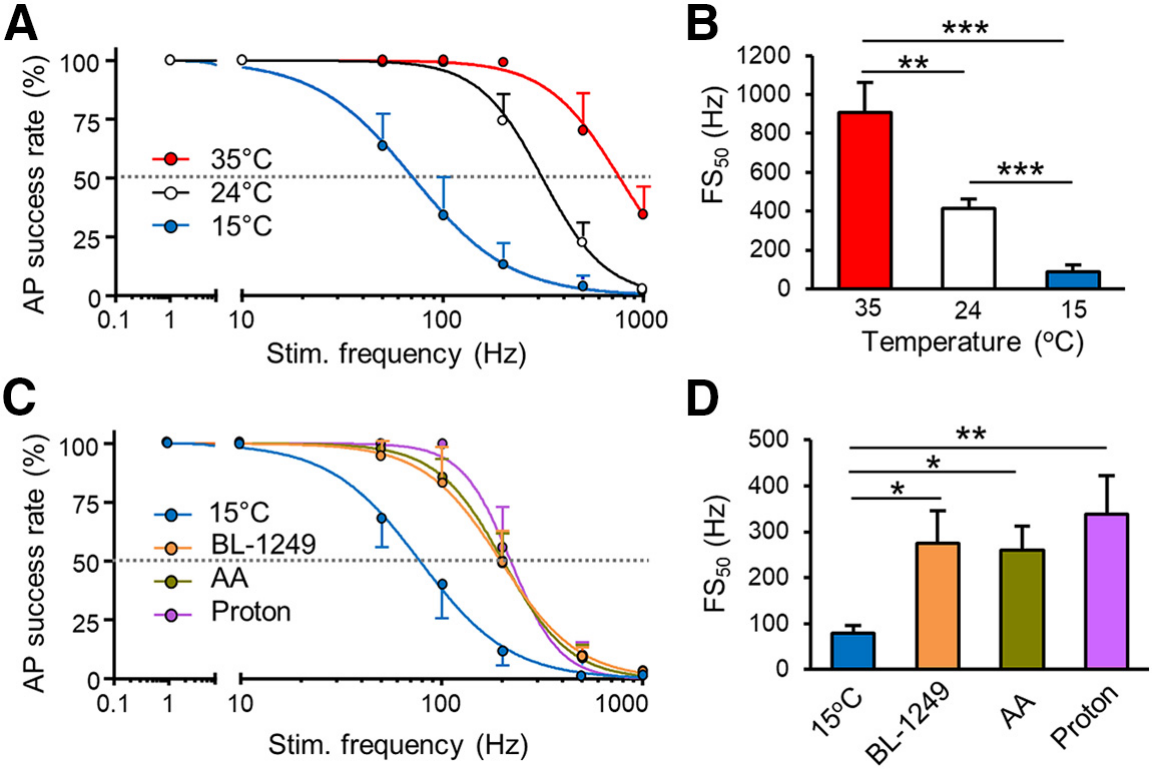
Temperature-dependent and frequency-dependent changes of AP success rate at the NR and effects of thermal K2P activators. ***A***, Plots of AP success rate against frequencies of train stimulation at 35°C (*n* = 6), 24°C (*n* = 6), and 15°C (*n* = 6). Train stimulation was applied at frequencies of 1, 10, 50, 100, 200, 500, and 1000 Hz each for a duration of 20 s. Dashed line indicates 50% AP success rate. ***B***, Bar graph shows summary data (*n* = 6) of FS_50_ values obtained from recordings performed at 35°C (*n* = 6), 24°C (*n* = 6), and 15°C (*n* = 6). FS_50_, frequencies at which the AP success rate is 50%. ***C***, Plots of AP success rate against different frequencies of train stimulation at 15°C in the absence (*n* = 6), presence of 10 μm BL (*n* = 6), 20 μm AA (*n* = 6), and protons (*n* = 5). Dashed line indicates 50% AP success rate. ***D***, Summary data of FS_50_ obtained from recordings at 15°C in the absence (*n* = 6), presence of BL (*n* = 6), AA (*n* = 6), and protons ([pH]_i_= 5, *n* = 5). Mean ± SEM, *p <* *0.05, **p <* *0.01, ***p <* *0.001, one-way ANOVA with the Tukey’s *post hoc* test.

We extended the above experiments by testing effects of cooling temperatures and thermal K2P activators on AP success rate following a broad range of stimulation frequencies. In this set of experiments, APs were elicited at distal sites of afferent nerves by train stimulation for 20 s at frequencies of 1, 10, 50, 100, 200, 500, and 1000 Hz. APs were recorded at NRs at three different temperatures, 35°C, 24°C, and 15°C. Figure 7*A* shows temperature-dependent and frequency-dependent changes of AP success rate as recorded at the NR. At 35°C, AP success rate was 100% for stimulation frequencies 1–200 Hz, and reduced to 70.9 ± 15.2% (*n* = 6) at train stimulation frequency of 500 Hz, and 35.1 ± 11.6% (*n* = 6) at train stimulation frequency of 1000 Hz. At 24°C, AP success rate versus stimulation frequency curve shifted to the left. AP success rate was 71.3 ± 13% at train stimulation frequency of 200 Hz, 23.5 ± 7.8% (*n* = 6) at train stimulation frequency of 500 Hz, and 0.97 ± 0.4% (*n* = 6) at train stimulation frequency of 1000 Hz. At 15°C, AP success rate versus train stimulation frequency curve further shifted left. AP success rate was 14 ± 8.6% (*n* = 6) at the train stimulation frequency of 200 Hz, 4.6 ± 4.2% (*n* = 6) at 500-Hz train stimulation, and 0.2 ± 0.2% (*n* = 6) at 1000-Hz train stimulation. We used the frequency at which AP success rate was 50% (FS_50_) to quantitatively describe the effects of temperatures and train stimulation frequency on AP success rates (Fig. 7*B*). FS_50_ was 910.1 ± 154.4 Hz (*n* = 6) at 35°C, and significantly reduced to 414.9 ± 51.9 Hz (*n* = 6, *p* < 0.01) at 24°C, and 89.1 ± 37.8 Hz (*n* = 6, *p* < 0.001) at 15°C (Fig. 7*B*). We further extended our study to quantitatively measure effects of thermal K2P activators on AP success rate at different stimulation frequencies at the cooling temperature of 15°C. As shown in Figure 7*C*, AP success rate versus train stimulation frequency curve at 15°C shifted to the right, i.e., higher frequency range, in the presence of K2P activators BL, AA, or protons (Fig. 7*C*). Quantitatively measured with FS_50_, the FS_50_ was 78.3 ± 16.4 Hz (*n* = 8) in the absence of the K2P activators, and increased to 275.7 ± 69.7 Hz (*n* = 6, *p* < 0.05) in the presence of 10 μm BL, 260.5 ± 51.1 Hz (*n* = 6, *p* < 0.05) in the presence of 20 μm AA, and 337.9 ± 84.1 Hz (*n* = 5, *p* < 0.01) in the presence of protons ([pH]_i_ = 5; Fig. 7*D*).

**Figure 8.**
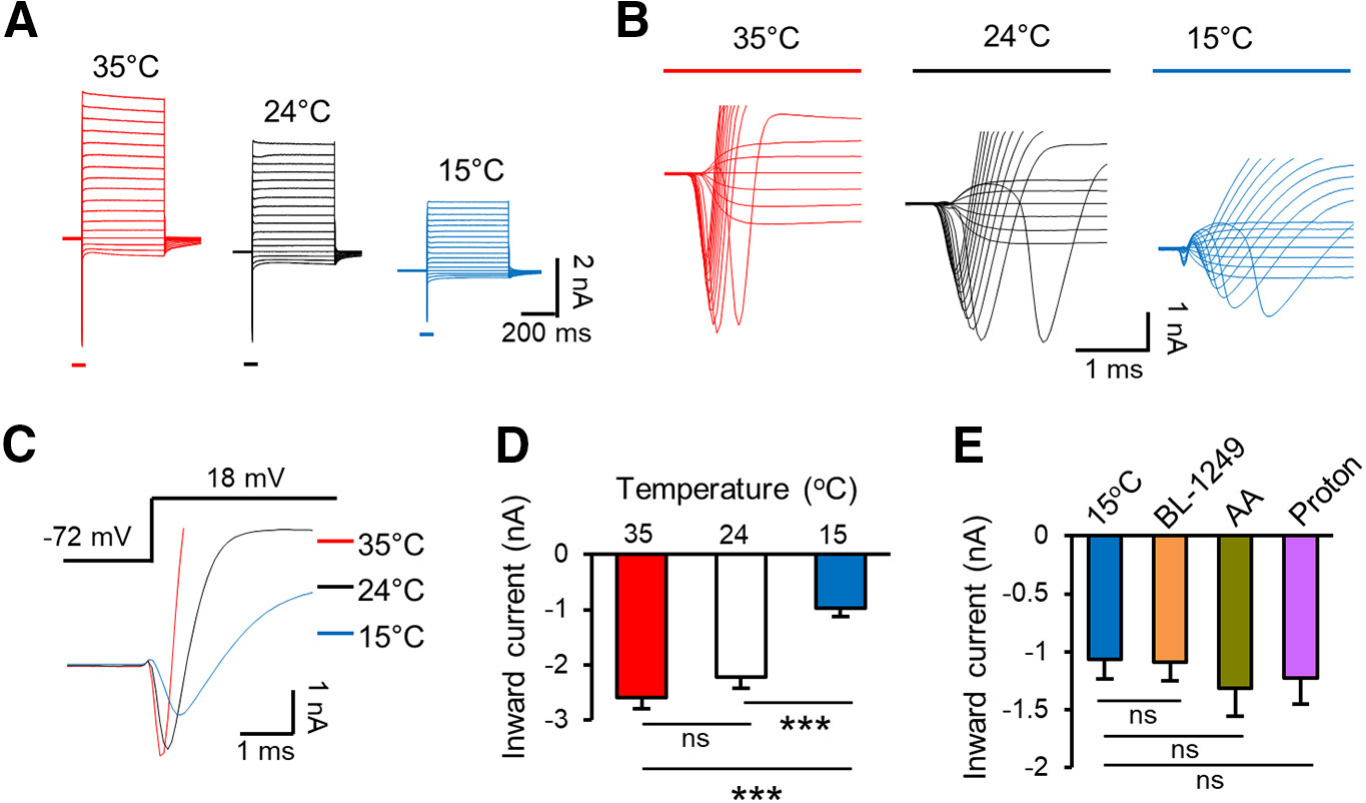
Effects of cooling temperatures and thermal K2P activators on voltage-gated Na^+^ current components at the NR. ***A***, Three sets of sample traces show inward and outward currents following series of voltage steps; the recordings were performed at 35°C (right), 24°C (middle), and 15°C (left). Nodal membranes were held at −72 mV. Voltage steps were applied from –102 to 58 mV with a 10-mV increment each step. ***B***, Three sets of sample traces show inward current components at an expanded scale with the recordings performed at 35°C (left), 24°C (middle), and 15°C (right). ***C***, Three overlay sample traces of inward current components evoked by a voltage step from −72 to 18 mV; the recordings were performed at 35°C (red), 24°C (black), and 15°C (blue). ***D***, Summary data of the amplitudes of inward current components recorded at 35°C (*n* = 8), 24°C (*n* = 8), and 15°C (*n* = 8). Voltage-step was from −72 to 18 mV. ***E***, Summary data of the amplitudes of inward current components recorded at 15°C in the absence (*n* = 6), presence of 10 μm BL (*n* = 7), 20 μm AA (*n* = 7), and protons ([pH 5]_i_, *n* = 8). Voltage-step was from −72 to 18 mV. Data represent mean ± SEM, * *p *<* *0.05, **p <* *0.01, ***p <* *0.001, one-way ANOVA with the Tukey’s *post hoc* test. ns, not significant.

The above experiments with thermal K2P activators demonstrate the partial involvement of thermal K2P channels in cooling temperature-induced reduction of AP success rate. Strong non-inactivating outward currents in response to depolarizing voltage steps were recorded from the NRs and the outward currents were substantially suppressed at the cooling temperature of 15°C (Fig. 8*A*). We have previously characterized the outward currents at NRs at different temperatures, and shown these currents were mediated by the thermal K2P channels TREK-1 and TRAAK and could be potentiated by BL, AA, or protons (Kanda et al., 2019). In addition to the non-inactivating outward currents (Fig. 8*A*), depolarizing voltage steps evoked transient inward currents (Fig. 8*A*,*B*) that were previously shown to be mediated by TTX-sensitive voltage-gated Na^+^ channels (Kanda et al., 2019). The inward currents were large and fast, often resulting in current escape from voltage-clamp commands near activation threshold (Fig. 8*B*). However, the inward currents evoked at higher voltage such as at the step from −72 to 18 mV showed no obvious escape from voltage-clamp as evidenced in the time course of the inward currents (Fig. 8*B*). Therefore, we chose the inward currents evoked by the step of −72–18 mV to quantitatively determine the effects of cooling temperatures on voltage-activated Na^+^ current components at the NRs (Fig. 8*C*,*D*). It should be noted that our voltage-clamp commands for recording the voltage-activate Na^+^ currents were approximate since it would be impossible to accurately voltage clamp the Na^+^ currents at the NR with a high resistance electrode. The amplitude of the inward current components was not significantly different between 35°C and 24°C (Fig. 8*C*,*D*). However, the inward current components measured at 15°C were significantly smaller in amplitudes and slower in kinetics than those measured at 35°C and 24°C (Fig. 8*D*). We next determined whether the inward current components at 15°C were affected by the thermal K2P activators. We found that the inward current components at NRs at 15°C were not significantly affected by 10 μm BL (*n* = 7), 20 μm AA (*n* = 7), or protons ([pH]_i_ = 5, *n* = 8). These results indicated that the effects of BL, AA, and protons on intrinsic electrophysiological properties and AP success rate were not because of potential non-specific effects on voltage-gated Na^+^ channels at the NR. It should be noted that although voltage-gated Na^+^ channels at the NR were significantly suppressed at the cooling temperature of 15°C (Fig. 8*D*), the AP amplitudes were significantly higher at 15°C than at 35°C (Fig. 2*B*,*D*).

### Cooling temperatures decrease availability of voltage-gated Na^+^ channels at depolarized NRs to impair high-frequency AP train regeneration

**Figure 9.**
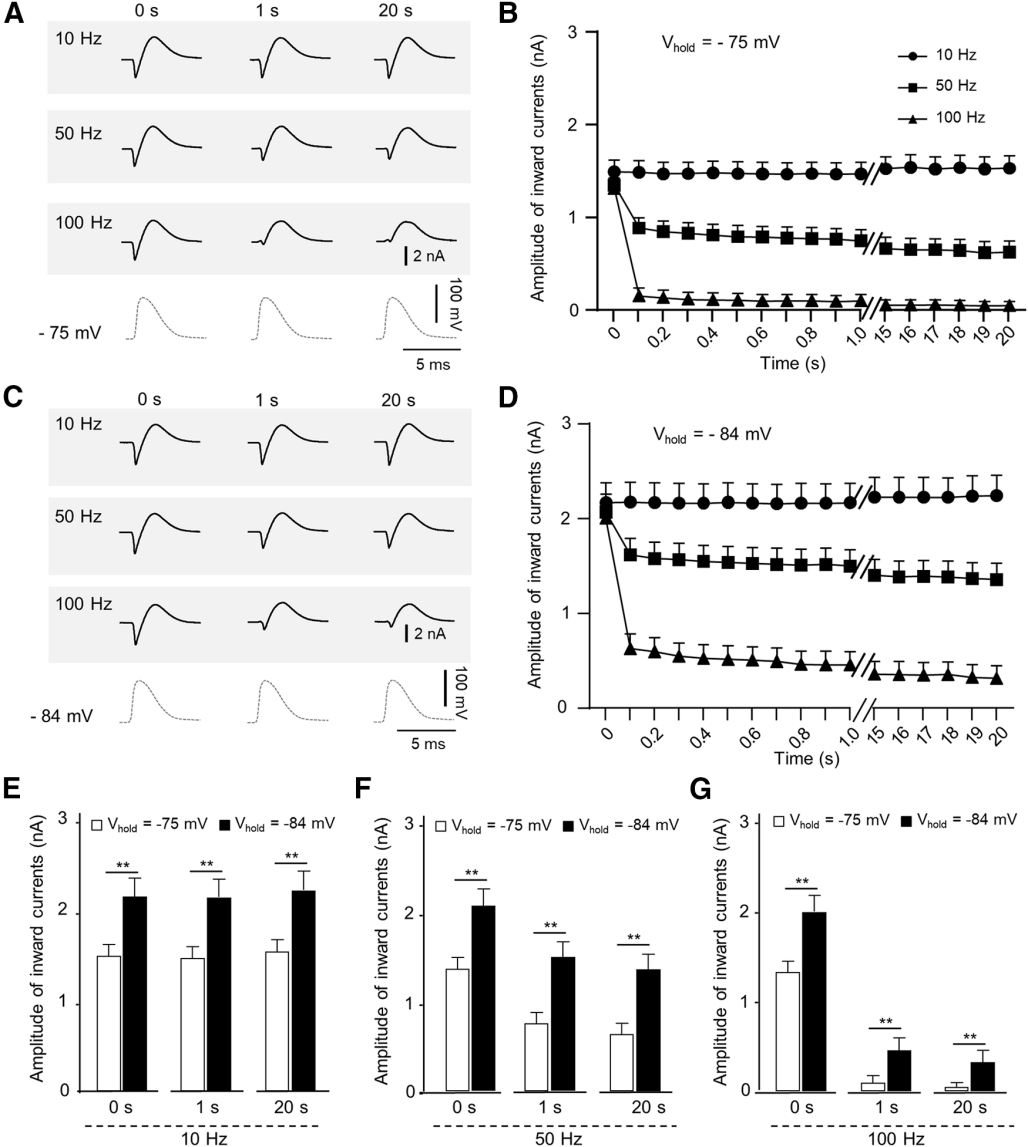
Membrane potential-dependent and frequency-dependent alterations of AP-activated Na^+^ currents at the NR at 15°C. ***A***, Three sets of sample traces of nodal membrane currents evoked by AP train stimulation at 10 Hz (first row), 50 Hz (second row), and 100 Hz (third row). Nodal membrane potentials were held at −75 mV and experiments performed at cooling temperature of 15°C. In each row, sample traces on left, middle, and right are the currents evoked at time points of 0 s (initial), 1 s, and 20 s, respectively. Traces in the fourth row are AP waveforms. In sample traces, AP-evoked voltage-gated Na^+^ currents are inward currents. Time scale: 5 ms. ***B***, Time course of amplitudes of inward currents evoked by AP train stimulation at 10 Hz (solid circles, *n* = 9), 50 Hz (solid squares, *n* = 9), and 100 Hz (solid triangles, *n* = 9). ***C***, ***D***, Similar to ***A***, ***B*** except membrane potentials were held at −84 mV. Inward currents were evoked by AP train stimulation at 10 Hz (*n* = 9, solid circles), 50 Hz (*n* = 9, solid squares), and 100 Hz (*n* = 9, solid triangles). ***E–G***, Comparison of amplitudes of inward currents with membrane potentials held at −75 mV (open bars) and −84 mV (solid bars). Data were from inward currents at time points of 0, 1, and 20 s. Inward currents were evoked by AP train stimulation at 10 Hz (*n* = 9, ***E***), 50 Hz (*n* = 9, ***F***), and 100 Hz (*n* = 9, ***G***). All recordings were performed at NRs at 15°C. Data represent mean ± SEM; ns, no significant difference, * *p *<* *0.05, **p <* *0.01, ***p <* *0.001, Student’s *t* test.

Cooling temperatures via thermal K2P channels caused substantial depolarization of RMPs at the NR (Figs. 1*F*, 3*E*). Depolarized NRs may promote steady-state inactivation of voltage-gated Na^+^ channels at cooling temperatures (Zimmermann et al., 2007). This would reduce the availability of voltage-gated Na^+^ channels for regeneration of high-frequency AP trains at the NR. To test this hypothesis, we applied AP-clamp recording technique (Liu et al., 2017) to the NR to investigate AP-activated Na^+^ currents in response to train stimulation with AP waveforms (AP train stimulation) at different frequencies (Fig. 9). In AP-clamp recordings, AP waveforms obtained from current-clamp recordings at the NR were applied as command voltages to the NR so that ionic currents during AP firing at the NR can be quantitatively determined. As exemplified in Figure 9*A*, application of AP waveforms to the NR evoked inward currents followed by outward currents. The inward currents were mediated by voltage-gated Na^+^ channels and outward currents by thermal K2P channels (Kanda et al., 2019). We performed AP-clamp recordings at 15°C with nodal membrane potentials held at −75 mV to determine how AP-activated Na^+^ currents were altered at different AP train stimulation frequencies (Fig. 9). The holding potential of −75 mV was selected in this set of experiments because it was approximately the RMP of the NR at 15°C (Fig. 1). As shown in Figure 9*A*,*B*, at 15°C and −75-mV holding potential, the inward currents were 1.493 ± 0.126 nA (*n* = 9) at initial time point (0 s) and remained unchanged to the end of 10-Hz AP train stimulation. However, with 50-Hz AP train stimulation, inward current amplitudes rapidly reduced by ∼50% and stayed reduced in the remaining AP train stimulation (Fig. 9*A*,*B*). Furthermore, with 100-Hz AP train stimulation, the inward current rapidly reduced becoming nearly negligible in the remaining 100-Hz AP train stimulation (Fig. 9*A*,*B*).

We next performed AP-clamp recordings at the NR at a holding potential of −84 mV to determine how a hyperpolarized membrane potential may affect AP-activated Na^+^ currents at 15°C. As shown in Figure 9*C*,*D*, inward currents were 2.175 ± 0.207 nA (*n* = 9) at initial time point (0 s) and remained unchanged to the end of 10-Hz AP train stimulation. With 50-Hz AP train stimulation, inward current amplitudes were reduced but remained relatively high (Fig. 9*C*,*D*). With 100-Hz AP train stimulation, inward currents were further reduced but remained at sizable amplitudes during 20 s AP train stimulation (Fig. 9*C*,*D*).

Comparing inward currents at 15°C between the AP-clamp recordings with holding potentials at −84 and at −75 mV, inward current amplitudes were significantly larger at the holding potential of −84 mV than at the holding potential of −75 mV for all three AP train stimulation frequencies tested (Fig. 9*G–I*). For example, with 10-Hz AP train stimulation, inward currents at 1-s time point were 2.163 ± 0.205 nA (*n* = 9) at the holding potential of −84 mV and 1.465 ± 0.123 nA (*n* = 9) at the holding potential of −75 mV (*p* < 0.01; Fig. 9*E*). With 50-Hz AP train stimulation, inward currents at 1-s time point were 1.495 ± 0.170 nA (*n* = 9) at the holding potential of −84 mV and 0.742 ± 0.120 nA (*n* = 9) at the holding potential of −75 mV (*p* < 0.01; Fig. 9*F*). With 100-Hz AP train stimulation, inward currents at 1-s time point were 0.324 ± 0.133 nA (*n* = 9) at the holding potential of −84 mV and only 0.095 ± 0.069 nA (*n* = 9) at the holding potential of −75 mV (*p* < 0.01; Fig. 9*G*). These results suggest that at the cooling temperature of 15°C, a depolarized nodal RMP partially contributes to lower availability of voltage-gated Na^+^ channels for high-frequency AP regeneration.

**Figure 10.**
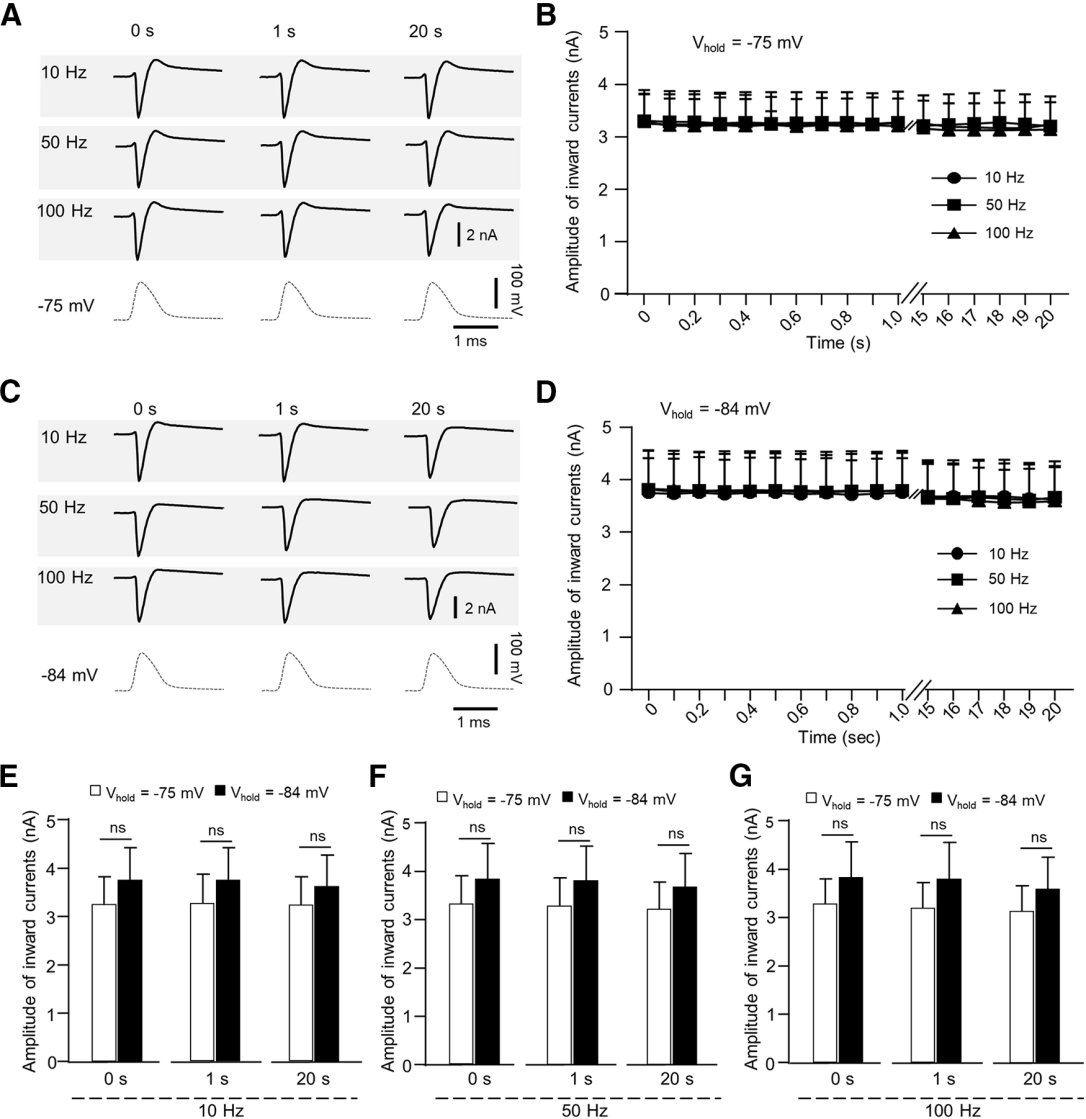
Lack of membrane potential-dependent and frequency-dependent alterations of AP-activated Na^+^ currents at the NR at 35°C. ***A***, Three sets of sample traces of nodal membrane currents evoked by AP train stimulation at 10 Hz (first row), 50 Hz (second row), and 100 Hz (third row). Nodal membrane potentials were held at −75 mV and experiments performed at 35°C. In each row, sample traces on left, middle, and right are the currents evoked at time points of 0 s (initial), 1 s, and 20 s, respectively. Traces in the fourth row are AP waveforms. Time scale: 1 ms. ***B***, Time course of amplitudes of inward currents evoked by AP train stimulation at 10 Hz (solid circles, *n* = 5), 50 Hz (solid squares, *n* = 5), and 100 Hz (solid triangles, *n* = 5). ***C***, ***D***, Similar to ***A***, ***B*** except membrane potentials were held at −84 mV. Inward currents were evoked by AP train stimulation at 10 Hz (*n* = 5, solid circles), 50 Hz (*n* = 5, solid squares), and 100 Hz (*n* = 5, solid triangles). ***E–G***, Comparison of amplitudes of inward currents at 35°C with membrane potentials held at −75 mV (open bars) and −84 mV (solid bars). Data were inward currents at time points of 0, 1, and 20 s. Inward currents were evoked by AP train stimulation at 10 Hz (*n* = 5), 50 Hz (*n* = 5), and 100 Hz (*n* = 5). All recordings were performed at NRs at 35°C. Data represent mean ± SEM; ns, no significant difference, Student’s *t* test.

We next determined the availability of voltage-gated Na^+^ channels at the NR at different AP train stimulation frequencies and different holding potentials at 35°C (Fig. 10). As shown in Figure 10*A*,*B*, at −75-mV holding potential, inward current amplitude was 3.248 ± 0.569 nA (*n* = 5) at initial time point (0 s) and remained unchanged during 10-Hz AP train stimulation. Inward current amplitudes during 50- and 100-Hz AP train stimulation were similar to those during 10-Hz AP train stimulation (Fig. 10*A*,*B*). At −84-mV holding potential, inward current amplitude was 3.750 ± 0.662 nA (*n* = 5) at initial time point (0 s) and remained unchanged during 10-Hz AP train stimulation (Fig. 10*C*,*D*). Inward current amplitudes during 50- and 100-Hz AP train stimulation was similar to those during 10-Hz AP train stimulation (Fig. 10*C*,*D*).

Comparing inward currents at 35°C between the recordings with holding potentials at −84 and at −75 mV, inward current amplitudes were not significantly different (Fig. 10*E–G*). For example, with 10-Hz AP train stimulation, inward currents at 1-s time point were 3.746 ± 0.665 nA (*n* = 5) at the holding potential of −84 mV, and 3.261 ± 0.595 nA (*n* = 5) at the holding potential of −75 mV (*p* = 0.30; Fig. 10*E*). With 50-Hz AP train stimulation, inward currents at 1-s time point were 3.795 ± 0.716 nA (*n* = 5) at the holding potential of −84 mV, and 3.279 ± 0.576 nA (*n* = 5) at the holding potential of −75 mV (*p* = 0.29; Fig. 10*F*). With 100-Hz AP train stimulation, inward currents at 1-s time point were 3.800 ± 0.749 nA (*n* = 5) at the holding potential of −84 mV, and 3.202 ± 0.523 nA (*n* = 5) at the holding potential of −75 mV (*p* = 0.27; Fig. 10*G*). These results indicate that, at 35°C, the availability of voltage-gated Na^+^ channels remain the same at AP train stimulation frequency up to 100 Hz at either holding potentials.

## Discussion

In the present study, we have shown that cooling temperatures significantly alter intrinsic electrophysiological properties at the NR of Aβ-afferent nerves. Furthermore, we have found that cooling temperatures severely compromise the regeneration of high-frequency AP trains at the NR. Importantly, we have demonstrated that thermal K2P channels at the NR are involved in cooling temperature-induced changes of intrinsic electrophysiological properties and regeneration of high-frequency AP trains at the NRs. Since tactile signals are mainly conveyed by Aβ-afferent nerves in trains of APs, our findings in the present study provide new insights into tactile signaling under cooling temperatures.

We have applied pressure-clamped patch-clamp recording technique in the present study (Kanda et al., 2019, 2021). This recording technique, developed recently, has allowed us to directly study ion channels and their functions at the NR (Kanda et al., 2019). Previously, our knowledge about ion channels at the NR is mainly from immunochemical studies (Rasband and Peles, 2021), which provides useful but limited information on functions of ion channels at the NR. Using pressure-clamped patch-clamp recordings, we have recently shown that the thermal K2P channels TREK1 and TRAAK are clustered at the NR of Aβ-afferent nerves to play a key role in AP repolarization, and high-speed and high-frequency saltatory conduction (Kanda et al., 2019). In the present study, our main focus was on how cooling temperatures impair regeneration of high-frequency AP trains at the NRs. Effects of cooling temperatures on AP conduction velocity at the NR have been reported in our previous study (Kanda et al., 2019). An interesting finding in the present study, consistent with our previous observation (Kanda et al., 2019), is that RMPs at the NR are hyperpolarized at approximately −84 mV at 35°C but are shifted to depolarized levels of approximately −75 mV at the cooling temperature of 15°C. In contrast, an earlier study has shown that cooling temperatures did not significantly affect RMPs of the giant axon of the squid (Hodgkin and Katz, 1949). In somatosensory neurons, previous studies show that cooling temperatures can depolarize, have no effect, or hyperpolarize RMPs depending on cell types (Cabanes et al., 2003; Kanda and Gu, 2017). These discrepancies may be because of whether thermal K2P channels are expressed on the nerves being tested. RMPs are known to be set mainly by outward leak K^+^ currents mediated by K2P channels (Enyedi and Czirják, 2010). In our recordings at the NR at 35°C, outward leak K^+^ currents were large in amplitude at the holding potentials of −72 mV. This is consistent with hyperpolarized RMPs at the NR at 35°C. On the other hand, outward leak K^+^ currents are suppressed at the cooling temperature of 15°C, which is accompanied by depolarization of RMP at the NR. These results support the idea that the effects of cooling temperatures on RMPs at the NR are via suppressing activities of thermal K2P channels. The involvement of thermal K2P channels in the depolarized RMPs at cooling temperatures is further supported by thermal K2P activators that reverse the effects of cooling temperatures. Similarly, the increases of membrane input resistance at cooling temperatures could also be attributed to the suppression of thermal K2P activity at the NR. For active membrane properties at the NR, we have shown that cooling temperatures broaden AP widths. AP widths are >2-fold broader at 15°C than at 35°C. Similarly, latencies to AP threshold are >2-fold longer at 15°C than at 35°C. The effects on AP widths and latencies to AP threshold by cooling temperatures are at least partially because of the suppression of thermal K2P activity since thermal K2P activator can partially reverse the effect of cooling temperatures. We have recently shown that TREK-1 and TRAAK, rather than voltage-gated K^+^ channels, are principal K^+^ channels involved in driving rapid AP repolarization (Kanda et al., 2019). Thus, suppression of these thermal K2P channels by cooling temperatures would reduce the driving force of AP repolarization leading to AP broadening.

In the present study, we show that cooling temperatures compromise the regeneration of high-frequency AP trains at the NR as is indicated by a decrease of AP success rate. The decrease of AP success rate by cooling temperatures occurs only when APs are elicited by high-frequency train stimulation. Consistently, cooling temperatures have been shown to impair regeneration of high-frequency AP trains in human ulnar nerves (Phillips and Matthews, 1993). One mechanism by which cooling temperatures can impair the regeneration of high-frequency AP trains at the NR is direct inhibition of voltage-gated Na^+^ channels by cooling temperatures. Cooling temperatures have been shown to directly slow kinetics and reduce amplitudes of voltage-activated Na^+^ currents in the somas of primary afferent nerves (Sarria et al., 2012), giant axons of squids (Matteson and Armstrong, 1982), and the NR of Aβ-afferent nerves as reported in the present study. In addition, subcellular distribution and membrane trafficking of voltage-gated Na^+^ channels (Bao, 2015) may be affected by cooling temperatures, which may be another contributing factor impairing regeneration of high-frequency AP trains at the NR. In the present study, however, the effects of cooling temperatures on regeneration of high-frequency AP trains at the NR were partially reversed by thermal K2P activators BL, AA and protons. These results suggest that the effects of cooling temperatures on the regeneration of high-frequency AP trains are at least partially mediated by thermal K2P channels. This raises the question of how cooling temperatures via thermal K2P channels affect regeneration of high-frequency AP trains. One contributing factor is that cooling temperatures suppress thermal K2P channels to broaden AP widths, which may prolong inter-spike intervals and reduce AP frequency at the NR. AP broadening leading to reduction of AP firing frequency has been observed in CNS neurons (Nomura et al., 2019). Another contributing factor is that cooling temperatures via suppressing thermal K2P channels prolong latency to AP threshold, which may also result in reduction of AP frequency at the NR. A third contributing factor is that cooling temperatures via suppressing thermal K2P channels depolarize RMPs, which in turn leads to the reduction of AP success rate. At cooling temperatures, a depolarized RMP would promote steady-state inactivation of voltage-gated Na^+^ channels and delay their recovery from inactivation (Kuo and Bean, 1994; Zimmermann et al., 2007). This would reduce the availability of voltage-gated Na^+^ channels for high-frequency AP trains. Consistently, at 15°C and with AP-clamp recording at the NR, inward currents were rapidly reduced following high-frequency AP waveform stimulation when nodal membranes were held at a depolarized level of −75 mV. On the other hand, when membrane potentials were held at a hyperpolarized level of −84 mV, inward currents were significantly higher in comparison with those with nodal membranes held at −75 mV. These results are consistent with previous studies showing that voltage-gated Na^+^ channels undergo steady-state inactivation at cooling temperatures in a membrane potential dependent manner (Zimmermann et al., 2007; Sarria et al., 2012). These results suggest that at cooling temperatures there is an interplay between RMP (via thermal K2P channels) and voltage-gated Na^+^ channels, which limits regeneration of high-frequency AP trains at the NR. A previous study has shown in cultured dorsal root ganglion neurons that at cooling temperatures there was an functional interplay between A-type K^+^ channels and voltage-gated Na^+^ channels to control AP firing (Sarria et al., 2012). In contrast to cooling temperatures, at 35°C inward currents were not significantly reduced during high-frequency AP waveform stimulation. This result indicates that at 35°C the voltage-gated Na^+^ channels can be recovered from inactivation rapidly during high-frequency AP trains.

Effects of cooling temperatures via thermal K2P channels on high-frequency AP regeneration at the NR and conduction along cutaneous Aβ-afferent nerves may have implications in sensory physiology and pathology. Exposing the skin to cooling environment, such as cold winter, can cool cutaneous Aβ-afferent nerves to low temperatures. While conduction block of myelinated nerves will occur at temperatures near or below 7°C (Franz and Iggo, 1968; Stecker and Baylor, 2009), impairment of regeneration of high-frequency AP train on myelinated nerves would happen at less cold temperatures (Franz and Iggo, 1968). Cutaneous Aβ-afferent nerves arising from low threshold mechanical receptors convey tactile signals with train APs in a broad range of frequencies. Tactile functions would be compromised if regeneration of high-frequency AP trains at the NR of Aβ-afferent nerves are impaired. Indeed, cooling temperatures can lead to poor acuity of tactile discrimination because of the impairment of regeneration of high-frequency AP trains on ulnar nerves at cooling temperatures in humans (Phillips and Matthews, 1993). In addition, the analgesic effect of cooling (Koç et al., 2006) may be partially attributed to the cooling-induced impairment of high-frequency AP trains in nociceptive A-afferent nerves. Cooling temperatures via thermal K2P channels on high-frequency AP regeneration and conduction on Aβ-afferent nerves may also have implications in sensory dysfunctions seen in patients with peripheral neuropathy.

## Synthesis

Reviewing Editor: Miriam Goodman, Stanford University Decisions are customarily a result of the Reviewing Editor and the peer reviewers coming together and discussing their recommendations until a consensus is reached. When revisions are invited, a fact-based synthesis statement explaining their decision and outlining what is needed to prepare a revision will be listed below. The following reviewer(s) agreed to reveal their identity: NONE. Note: If this manuscript was transferred from JNeurosci and a decision was made to accept the manuscript without peer review, a brief statement to this effect will instead be what is listed below.

Thank you for forwarding this manuscript to eNeuro. Your responses to the prior critiques addresses their main concerns. One concern is only partly addressed, which is the advance relative to Kanda et al, 2019. In this prior study, you showed that TREK-1 and TRAAK localize to NR and play a central role in temperature-dependent repolarizing. This study extends these findings to address temperature-dependence of firing rate and the privileged role of these channels in this phenomonen. To make this advance more salient to readers, please consider revising the introduction to reference prior work more thoroughly and to highlight the questions that were raised but unanswered. You might also consider speculating in the discussion and significant statement that the analgesic effect of cooling is due to the role of these channels at NR.

eNeuro is recommending that authors consider shifting statistical analyses to using estimation statistical approaches. Your manuscript is a good candidate for this approach (e.g. all figures that include bar graphs). Please also consider showing all of the individual data points along with the mean{plus minus}ERR in the bar graphs. This approach would allow readers to get a more fullsome understanding of the variation in the findings.

Thank you!
